# NCAPG confers trastuzumab resistance via activating SRC/STAT3 signaling pathway in HER2-positive breast cancer

**DOI:** 10.1038/s41419-020-02753-x

**Published:** 2020-07-18

**Authors:** Lili Jiang, Liangliang Ren, Han Chen, Jinyuan Pan, Zhuojun Zhang, Xiangqin Kuang, Xuhong Chen, Wenhao Bao, Chun Lin, Zhongqiu Zhou, Danping Huang, Jianan Yang, Hongbiao Huang, Lan Wang, Ning Hou, Libing Song

**Affiliations:** 1 Affiliated Cancer Hospital & Institute of Guangzhou Medical University, 510095 Guangzhou, China; 2 Guangzhou Municipal and Guangdong Provincial Key Laboratory of Protein Modification and Degradation, School of Basic Medical Science, Guangzhou Medical University, 511436 Guangzhou, China; 3 Department of Biochemistry, Zhongshan School of Medicine, Sun Yat-sen University, 510080 Guangzhou, China; 4 Department of Ultrasonography, Guangzhou Women and Children’s Medical Center, Guangzhou Medical University, 510623 Guangzhou, China; 5 Department of Urologic Oncosurgery, Affiliated Cancer Hospital & Institute of Guangzhou Medical University, 510095 Guangzhou, China; 6 Department of Pathogen Biology and Immunology, School of Basic Courses, Guangdong Pharmaceutical University, 510006 Guangzhou, China; 7 Key Laboratory of Molecular Target & Clinical Pharmacology, School of Pharmaceutical Sciences & the Fifth Affiliated Hospital, Guangzhou Medical University, 511436 Guangzhou, China; 8 State Key Laboratory of Oncology in South China, Collaborative Innovation Center for Cancer Medicine, Sun Yat-sen University Cancer Center, 510060 Guangzhou, China

**Keywords:** Cancer, Cell biology

## Abstract

HER2+ breast cancer (BC) is characterized by rapid growth, early recurrence, early metastasis, and chemoresistance. Trastuzumab is the most effective treatment for HER2+ BC and effectively reduces the risk of recurrence and death of patients. Resistance to trastuzumab results in cancer recurrence and metastasis, leading to poor prognosis of HER2+ BC. In the present study, we found that non-structural maintenance of chromosome condensin 1 complex subunit G (NCAPG) expression was highly upregulated in trastuzumab-resistant HER2+ BC. Ectopic NCAPG was positively correlated with tumor relapse and shorter survival in HER2+ BC patients. Moreover, overexpression of NCAPG promoted, while silencing of NCAPG reduced, the proliferative and anti-apoptotic capacity of HER2+ BC cells both in vitro and in vivo, indicating NCAPG reduces the sensitivity of HER2+ BC cells to trastuzumab and may confer trastuzumab resistance. Furthermore, our results suggest that NCAPG triggers a series of biological cascades by phosphorylating SRC and enhancing nuclear localization and activation of STAT3. To summarize, our study explores a crucial role for NCAPG in trastuzumab resistance and its underlying mechanisms in HER2+ BC, and suggests that NCAPG could be both a potential prognostic marker as well as a therapeutic target to effectively overcome trastuzumab resistance.

## Introduction

Breast cancer (BC) remains the leading cause of morbidity and mortality among cancers in females worldwide^1^. Based on gene expression profiling and molecular pathology, BC was traditionally classified into luminal A, luminal B, human epidermal growth factor receptor 2 positive (HER2+), and Basal-like type subtypes^2,3^. HER2+ BC accounts for 15–20% of total cases of BC, and is characterized by a high degree of malignancy and strong invasiveness, high resistance to chemotherapy, poor effectiveness of endocrine therapy, early recurrence and metastasis, and poor prognosis^4,5^.

Trastuzumab, a humanized HER2-targeting drug, is the most effective treatment for HER2+ BC. Trastuzumab effectively reduces the risk of recurrence and death, extends the disease-free and non-progressive survival period, delays the recurrence time of metastasis, and prolongs survival particularly in patients with metastasis^6,7^. However, the resistance rate of trastuzumab is 66–88% as a single agent and 20–50% when combined with chemotherapy in HER2+ BC, and trastuzumab resistance is the major cause of treatment failure resulting in relapse or death^8,9^. Therefore, it is critical to investigate the molecular mechanisms underlying trastuzumab resistance in HER2+ BC in order to improve therapeutic effectiveness and prognosis of patients.

Non-structural maintenance of chromatin condensin 1 complex subunit G (NCAPG) is a subunit of condensin 1. Human NCAPG was purified initially from HeLa cell nuclear extracts and plays a vital role in condensin activation by regulation of ATPase activity^10,11^. As a tumor-promoting gene, NCAPG has been found overexpressed in several malignancies, including prostate cancer, pediatric high-grade gliomas, and hepatocellular carcinoma (HCC)^12–14^. Upregulation of NCAPG has been suggested to facilitate cell proliferation in gastric cancer^15^. Moreover, ectopic NCAPG was shown to promote cell proliferation, metastasis, differentiation in HCC and was associated with poor TNM stages, poor survival, and tumor recurrence in HCC patients^16,17^. Although NCAPG has previously been reported to be overexpressed and correlated with poor survival in BC^18,19^, its biological role and mechanism have not been explored.

In the present study, we found that NCAPG was significantly overexpressed in trastuzumab-resistant HER2+ BC samples and upregulation of NCAPG correlated with poor survival and relapse in HER2+ BC patients. Overexpression of NCAPG was found to promote cell proliferation and decrease trastuzumab-induced apoptosis, while silencing NCAPG re-sensitized resistant HER2+ BC cells to trastuzumab. Furthermore, the SRC/STAT3 pathway was identified to be activated with NCAPG overexpression and mediated trastuzumab resistance-related cellular functions. These results reveal a critical role for NCAPG in conferring trastuzumab resistance and suggest that NCAPG may be a potential prognostic factor, and therapeutic target, against trastuzumab resistance in HER2+ BC.

## Results

### NCAPG is upregulated in trastuzumab-resistant BC

**Fig. 1:**
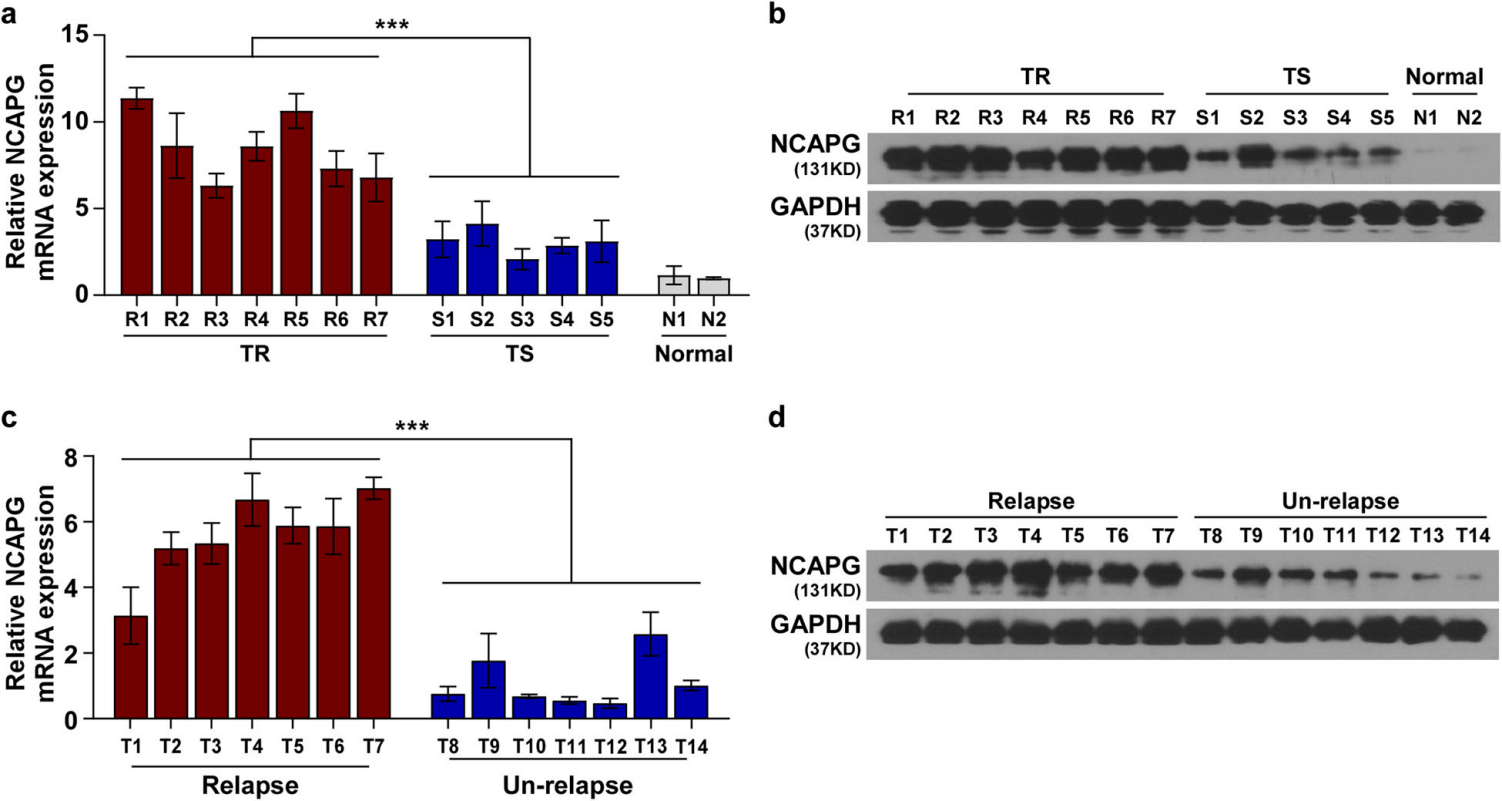
NCAPG is overexpressed in trastuzumab-resistant breast cancer. **a**, **b** NCAPG expression was analyzed by qPCR **a** and western blotting **b** in two normal breast tissues and 12 breast cancer specimens, including seven samples of trastuzumab-resistant (TR) and five samples of trastuzumab-sensitive (TS) patients. **c**, **d** qPCR **c** and western blotting results **d** of NCAPG expression in BC tissues from patients with or without relapse. Each bar represents the mean ± SD of three independent experiments. ***P < 0.001.

The Cancer Genome Atlas (TCGA) data analysis displayed that NCAPG was markedly upregulated in BC tissues, particularly in HER2+ cancer tissues (*P* < 0.001) (Fig. S1a, b). To further investigate clinical significance and biological role of NCAPG in the trastuzumab-resistant BC, its expression level in HER2+ BC biopsies with different tolerance to trastuzumab treatment was examined. The results showed that NCAPG was significantly upregulated in trastuzumab-resistant biopsies compared to trastuzumab-sensitive and normal tissues (Fig. 1a, b). NCAPG expression was also significantly increased in the trastuzumab-resistant BC cell lines SKBR3/TR and BT474/TR compared to parental SKBR3 and BT474 cell lines (Fig. S2). As trastuzumab resistance results in higher tumor relapse rates of HER2+ BC^20^, we next assessed the expression of NCAPG in patients with different relapse status. Both the levels of NCAPG mRNA and protein were remarkably increased in freshly collected BC tissues from patients with tumor relapse compared to patients without relapse (Fig. 1c, d). These findings suggest that NCAPG upregulation is associated with, and may contribute to, the promotion of trastuzumab resistance in HER2+ BC.

### High NCAPG expression correlates with poor prognosis in BC patients

**Fig. 2:**
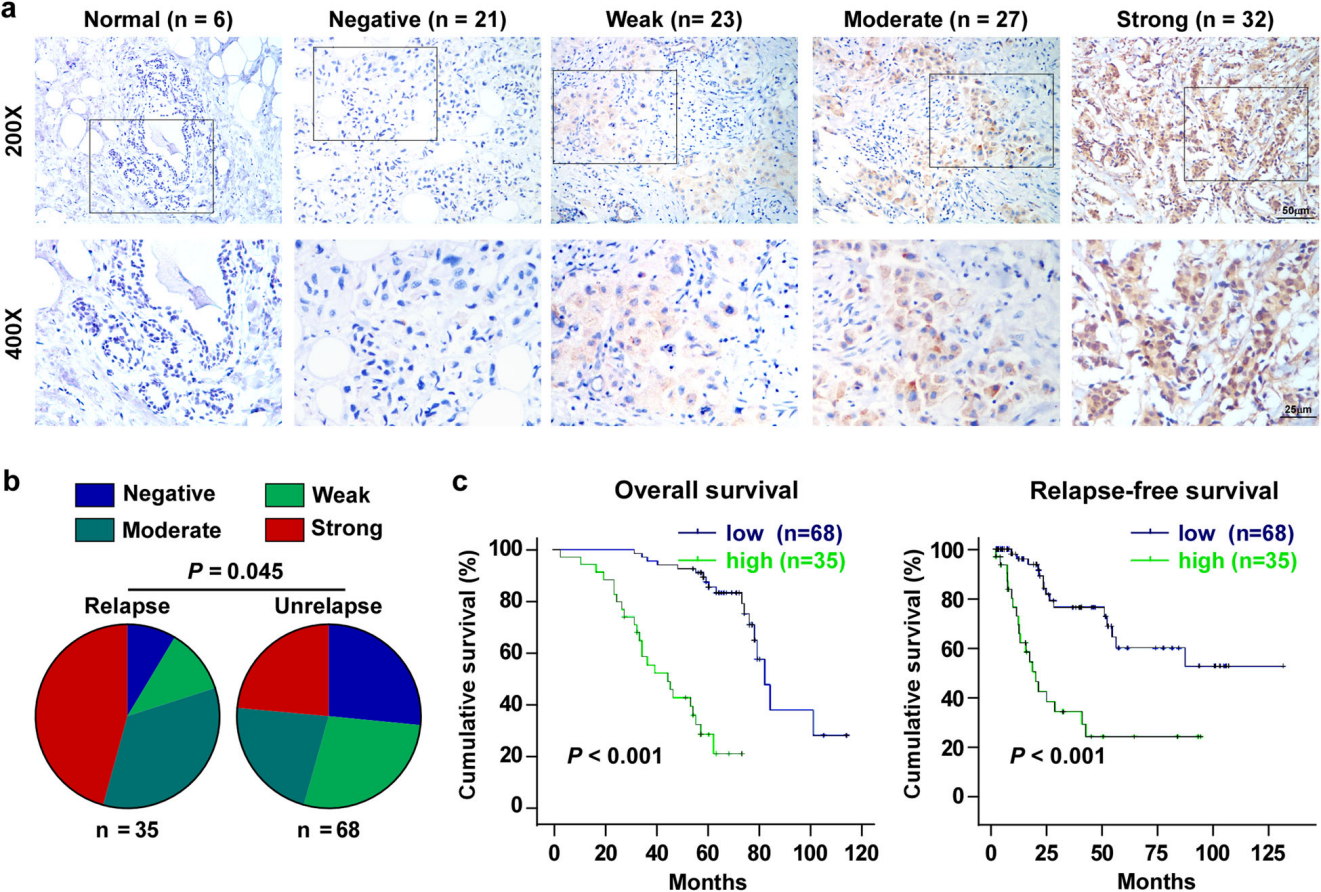
NCAPG correlates with poor prognosis in breast cancer. **a** Representative IHC images of NCAPG staining in six normal breast tissues and 103 breast specimens, which were scored as negative (0), weak (+1), moderate (+2), and strong (+3). **b** Distribution and comparison between NCAPG-staining grade and tumor relapse in breast cancer patients. *χ*^2^ test was used. **c** Kaplan–Meier analysis of overall survival and relapse-free survival curves stratified by low and high NCAPG expression in BC patients treated with trastuzumab (*n* = 103, log-rank test). Error bar represents the mean ± SD of three independent experiments.

Immunohistochemistry (IHC) was used to assess NCAPG expression in 103 BC tissue specimen with trastuzumab therapy and six normal paraffin-embedded breast tissues (Supplementary Table S1). NCAPG expression was low or nearly non-detectable in all normal breast tissues (Fig. 2a). In contrast, BC tissues specimen revealed a broader spectrum of expression. Levels of NCAPG were negative (0) in 21 cases, weak (+1) in 23, moderate (+2) in 27, and strong (+3) in 32 cases. A correlation analysis showed that NCAPG expression was positively correlated with the age (*P* = 0.009), clinical stage (*P* = 0.003), tumor size (T classification, *P* = 0.022), metastasis (M classification, *P* = 0.014), vital status (*P* < 0.001), as well as the relapse status (*P* < 0.05) (Fig. 2b, Supplementary Table S2). In addition, the Kaplan–Meier survival curves and log-rank tests indicated that patients of NCAPG overexpression had a significantly poorer overall survival (*P* < 0.001) and relapse-free survival (*P* < 0.001) (Fig. 2c). Consistently, the analysis resulting from the public database Kaplan–Meier Plotter^21^ (http://kmplot.com/analysis) revealed that BC patients of higher NCAPG expression were correlated significantly with poorer overall, distant metastasis-free and relapse-free survival (Fig. S3). Univariate (*P* < 0.001) and multivariate (*P* = 0.013) analyses showed that NCAPG was an independent prognostic factor in patients with trastuzumab-resistant BC (Supplementary Table S3). To summarize, NCAPG may represent a potential unfavorable prognostic marker in trastuzumab-resistant BC.

### NCAPG knockdown re-sensitizes trastuzumab-resistant BC cell in vivo

**Fig. 3:**
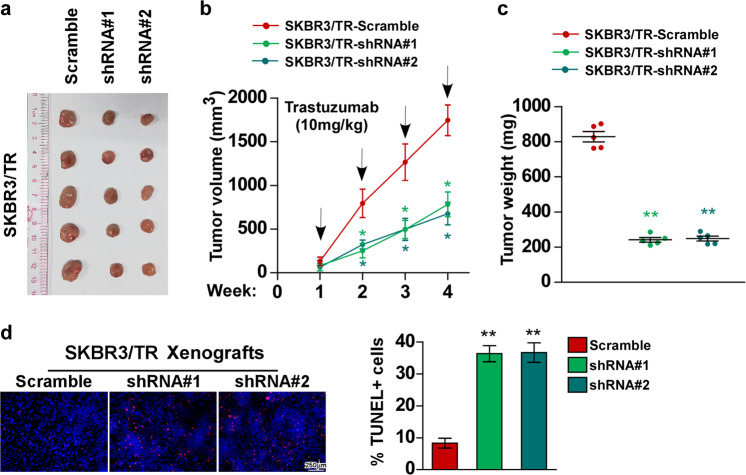
Knockdown of NCAPG sensitizes resistant HER2+ BC cells to trastuzumab in vivo. **a** SKBR3/TR-Scramble, SKBR3/TR-shRNA#1, and SKBR3/TR-shRNA#2 were subcutaneously injected into BALB/c nude mice (*n* = 5 mice/group). When the average size of the tumor reached 100 mm^3^, the mice were treated with an intraperitoneal injection of trastuzumab (10 mg/kg, once a week) for 4 weeks. **b** The tumor volumes were examined weekly. **c** Tumor weights. **d** Representative fluorescence images (left) and quantification (right) of TUNEL-stained cells in xenografts formed by the indicated cells treated with trastuzumab. A two-tailed *t-*test was used for the statistical analysis. Error bar represents the mean ± SD of three independent experiments. *P < 0.05; **P < 0.01.

To explore the biological function of NCAPG in trastuzumab-resistant BC cells, NCAPG was silenced in the trastuzumab-resistant BC cell line SKBR3/TR using two independent short-hairpin constructs (Fig. S4a, b). Xenograft experiment was used to investigate the role of NCAPG in trastuzumab resistance in vivo. SKBR3/TR and NCAPG-silenced SKBR3/TR cells were subcutaneously administered into nude mice. During treatment with trastuzumab, the tumors formed by SKBR3/TR cells retained a higher growth rate, revealing a considerable degree of trastuzumab resistance (Fig. 3a–c). In contrast, the capacity of tumor growth by cells with NCAPG silencing (SKBR3/TR-shRNA#1/2) was strongly hampered in mice, indicating that NCAPG expression was crucial for trastuzumab resistance in SKBR3/TR cells. Simultaneously, the pro-apoptotic activity of trastuzumab was enhanced by silencing NCAPG (Fig. 3d). Thus, these results indicate that silencing NCAPG re-sensitizes resistant HER2+ BC cells towards trastuzumab therapy.

### NCAPG confers trastuzumab resistance in HER2+ BC cells in vitro

**Fig. 4:**
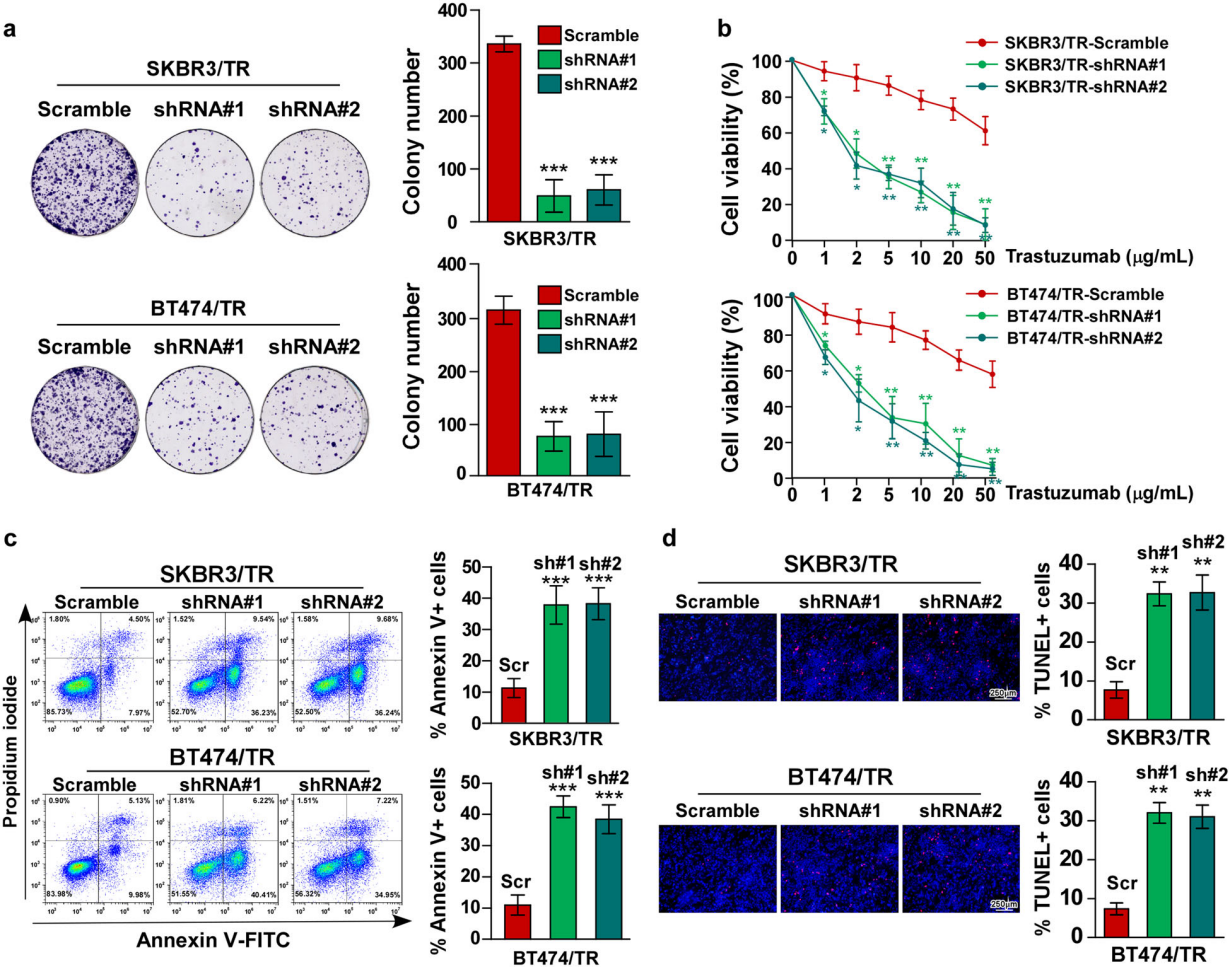
Knockdown of NCAPG sensitizes resistant HER2+ BC cells to trastuzumab in vitro. **a** Representative micrographs (left) and quantification (right) of colony formation assays for trastuzumab-resistant SKBR3 and BT474 cell lines, SKBR3/TR and BT474/TR, with or without NCAPG knockdown under treatment with trastuzumab (10 μg/mL). **b** Cell viability measured by MTT assay. **c** Flow cytometry analysis of apoptosis by Annexin V-FITC/PI staining (left) and histograms (right), indicating the percentage of Annexin V+ cells. **d** Representative images (left) and quantification (right) of fluorescence micrographs TUNEL-stained cells in the indicated cells treated with trastuzumab (10 μg/mL). Scr Scramble; sh shRNA. Error bar represents the mean ± SD of three independent experiments. *P < 0.05; **P < 0.01; ***P < 0.001.

**Fig. 5:**
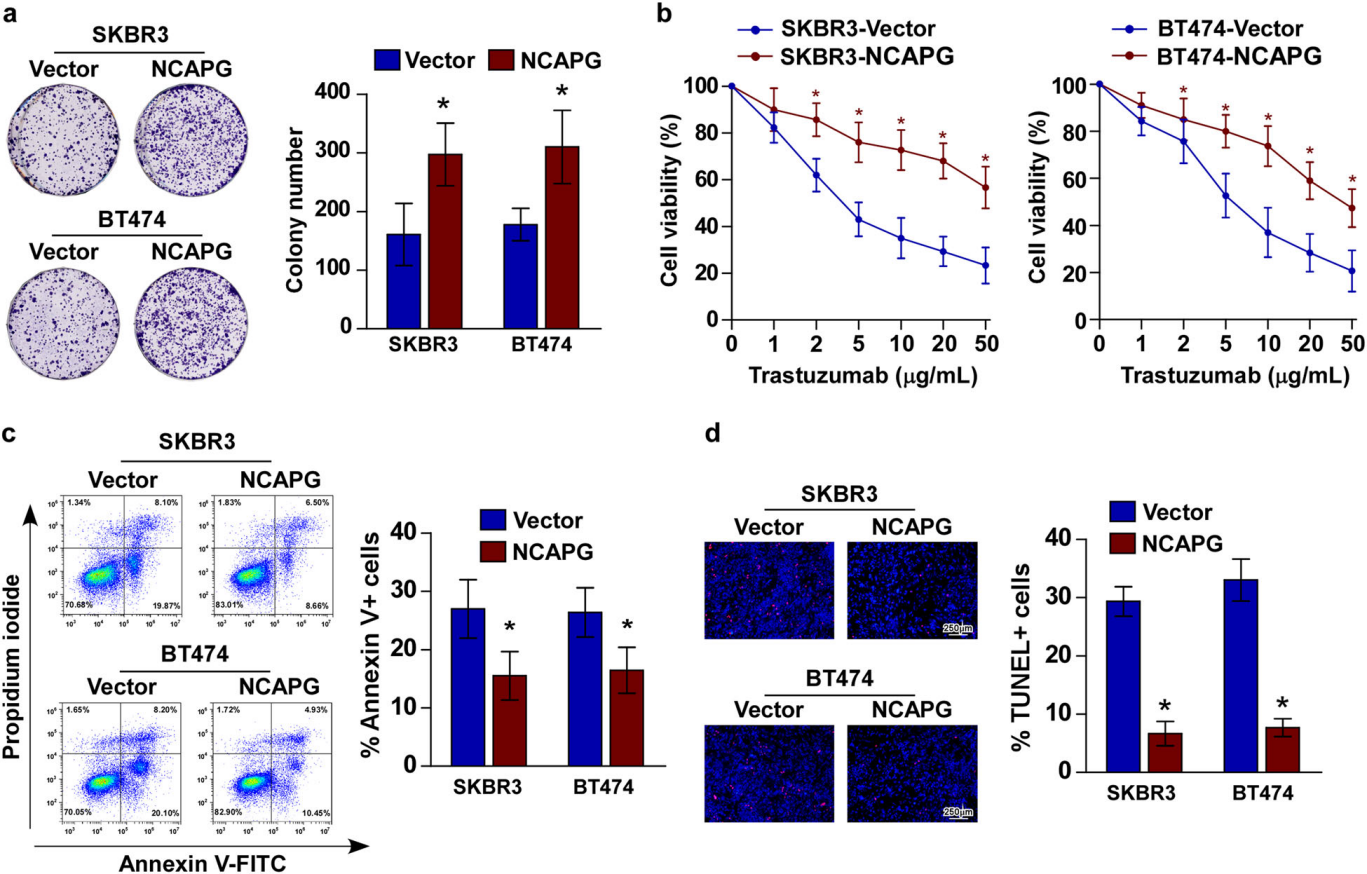
Ectopic NCAPG renders trastuzumab resistance in breast cancer. **a** Representative micrographs (left) and quantification (right) of colony formation assays for SKBR3 and BT474 cell lines with or without overexpression of NCAPG under treatment with trastuzumab (10 μg/mL). **b** Cell viability measured by MTT assay. **c** Flow cytometry analysis of apoptosis by Annexin V-FITC/PI staining (left) and histograms (right) indicating the percentage of Annexin V+ cells. **d** Representative images (left) and quantification (right) of fluorescence micrographs of TUNEL-stained cells in the indicated cells treated with trastuzumab (10 μg/mL). Error bar represents the mean ± SD of three independent experiments. *P < 0.05.

The biological role of NCAPG in BC resistance to trastuzumab was also investigated in vitro. Colony formation and cell viability analyses revealed that knockdown NCAPG re-sensitizes SKBR3/TR and BT474/TR cells to trastuzumab treatment (Fig. 4a, b). As observed in vivo, the pro-apoptotic activity of trastuzumab treatment was increased in SKBR3/TR and BT474/TR cells with silenced NCAPG expression (Fig. 4c, d). Furthermore, the cell lines stably overexpressing NCAPG, SKBR3–NCAPG, and BT474–NCAPG, were established for further study (Fig. S5). Consistent with the previous results, the proliferative ability of SKBR3–NCAPG and BT474–NCAPG was significantly enhanced, even after trastuzumab treatment, compared to control cells (Fig. 5a, b). Moreover, Annexin V/FITC and TUNEL staining assays both showed that trastuzumab-induced apoptosis of SKBR3 and BT474 cells was significantly reduced by NCAPG overexpression (Fig. 5c, d). These findings indicate that ectopic NCAPG expression may confer trastuzumab resistance in BC cells.

### NCAPG activates SRC/STAT3-signaling pathway

**Fig. 6:**
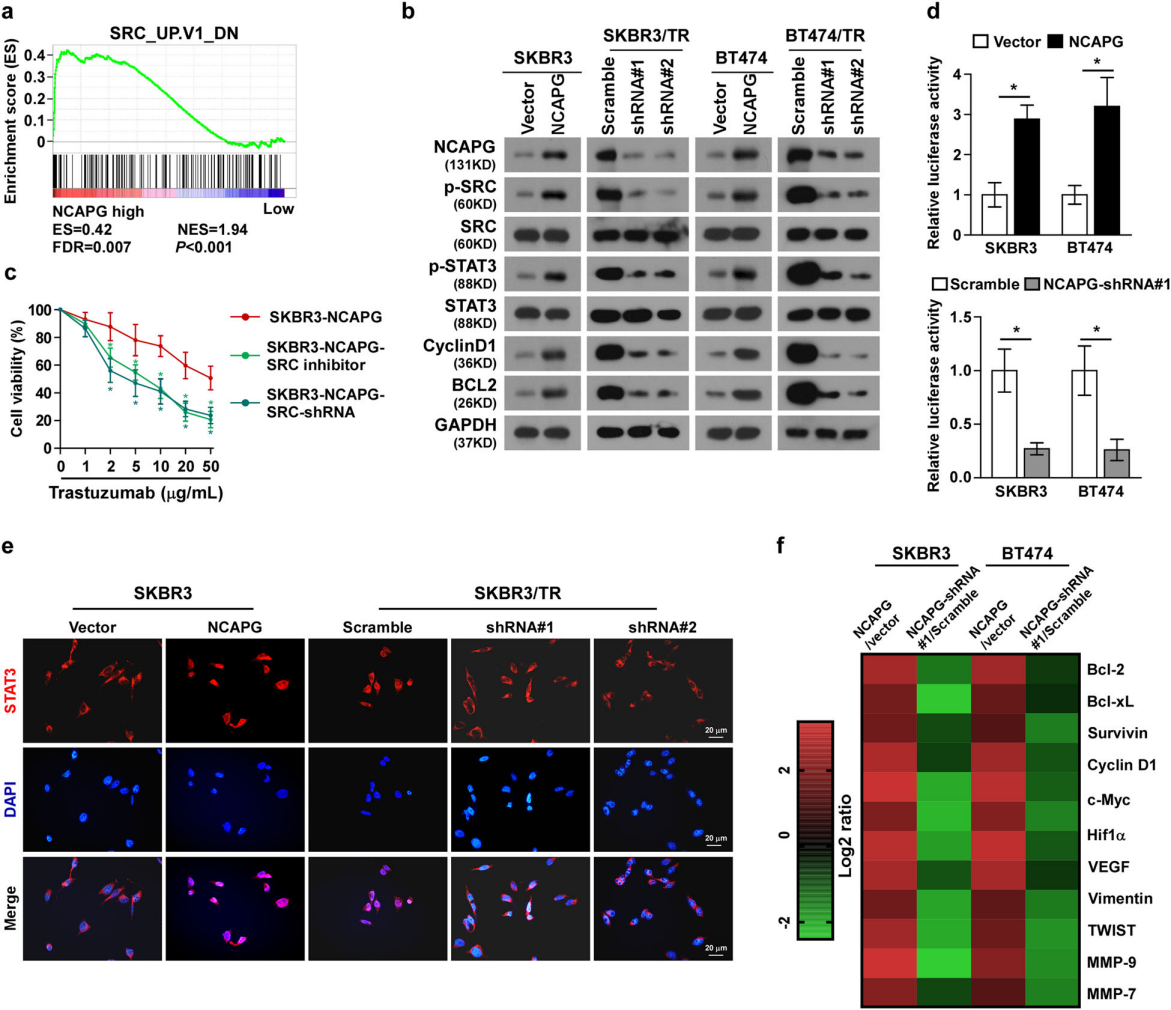
NCAPG activates SRC/STAT3-signaling pathway. **a** Gene set enrichment analysis (GSEA) in the published breast cancer dataset showed the level of NCAPG expression was correlated with the gene signatures (SRC_UP. V1_DN). **b** Western blotting analysis of the expressions of p-SRC, SRC, p-STAT3, STAT3, CyclinD1, and BCL2, in indicated cells. **c** Cell viability measured by MTT assay. **d** Relative luciferase activity of STAT3 in indicated cells. **e** Fluorescence immunostaining revealed the subcellular localization of STAT3 in the indicated cells. **f** Heat map showing NCAPG promotes the mRNA expression of STAT3 downstream genes. Error bar represents the mean ± SD of three independent experiments. *P < 0.05.

To investigate the molecular mechanism underlying the ability of NCAPG to confer trastuzumab resistance in BC, gene set enrichment analysis (GSEA) in the published BC dataset was performed. As shown, the expression level of NCAPG was positively correlated with SRC-related gene signatures, suggesting NCAPG might be involved in activation of SRC signaling (Fig. 6a). In line with this hypothesis, western blotting showed that NCAPG overexpression increased, while silencing NCAPG decreased, phosphorylation of SRC (Tyr416), indicating NCAPG promotes SRC signaling activation (Fig. 6b). Accordingly, the promotion of the proliferative capacity of cells by NCAPG was weakened by inhibiting SRC using a specific inhibitor or shRNA (Fig. 6c), indicating that SRC activity is essential for NCAPG-dependent trastuzumab resistance. The transcription factor STAT3 is downstream of the SRC pathway, and has previously been shown to be correlated with drug resistance of tumors^22^. Therefore, the activity of STAT3 was also examined. The transcriptional activity of STAT3 was enhanced in NCAPG-overexpressing cells, while it was inhibited in NCAPG-silenced cells (Fig. 6d). Simultaneously, ectopic NCAPG increased, while silencing NCAPG decreased, the phosphorylation of STAT3, as well as the expression of STAT3-signaling downstream factors, Cyclin D1 and BCL2, which are markers for cell proliferation and anti-apoptotic capacity, respectively (Fig. 6b). Furthermore, the nuclear localization of STAT3 was found to be promoted by NCAPG overexpression and suppressed by NCAPG silencing (Figs. 6e and S6). The mRNA expression of STAT3 downstream genes was also upregulated by NCAPG (Fig. 6f). Collectively, these results suggest that NCAPG exerts trastuzumab resistance through activation of the SRC/STAT3-signaling pathway.

### NCAPG expression correlates clinically with the activation of SRC/STAT3 signaling in BC

**Fig. 7:**
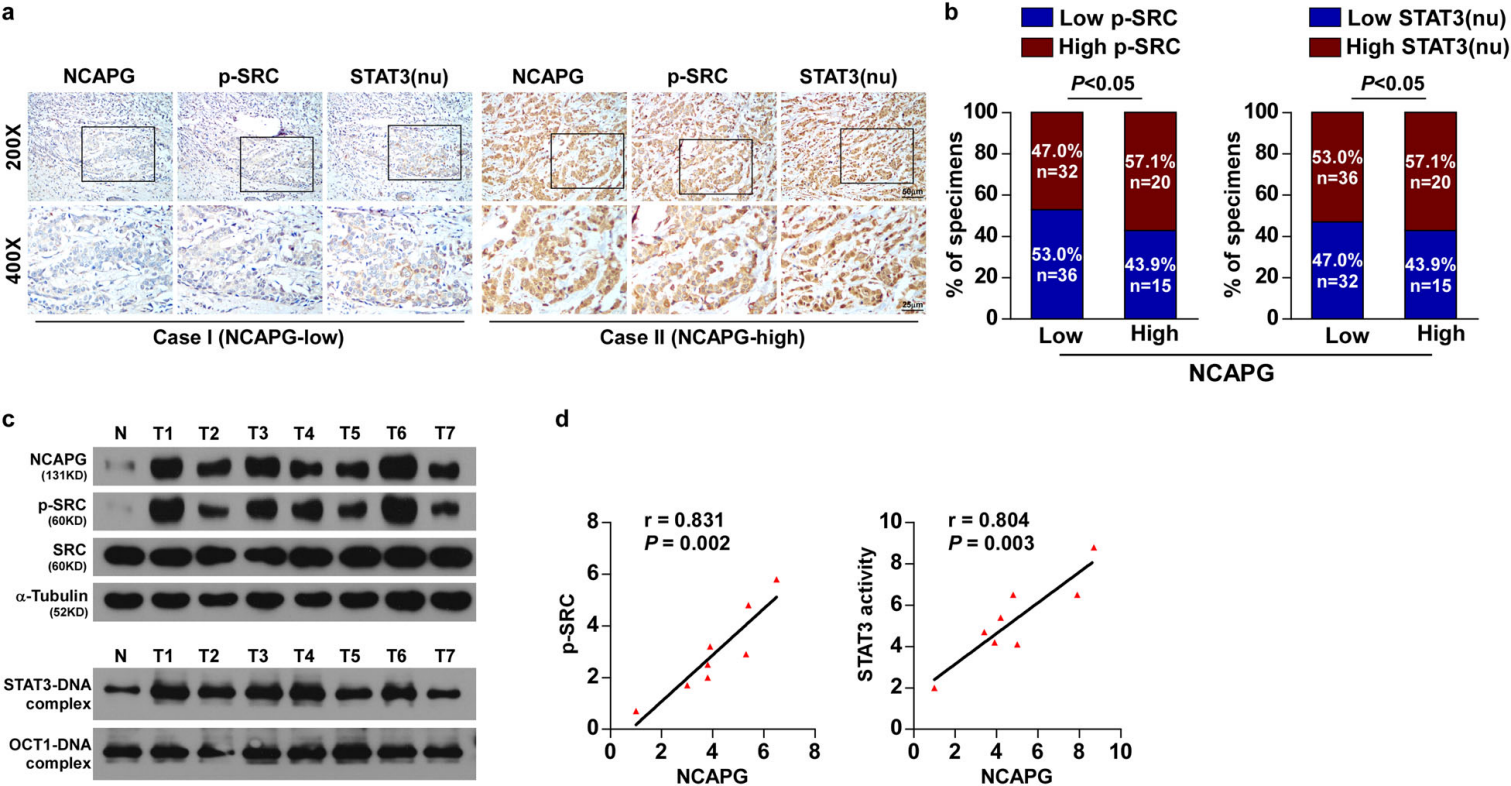
NCAPG expression correlates clinically with the activation of SRC/STAT3 signaling in breast cancer. **a** Representative IHC images of SRC, p-SRC, nuclear STAT3 with low and high expressions of NCAPG. **b** Distribution and comparison between NCAPG expression and p-SRC and nuclear STAT3 in breast cancer patients. nu nuclear. **c** Western blotting detection of NCAPG expression, EMSA analysis of STAT3 DNA-binding activity in seven freshly collected human breast cancer samples, compared with one normal breast tissue. **d** Correlation analyses of NCAPG expression with p-SRC expression and STAT3 DNA-binding activity.

Analysis of IHC staining revealed that enhanced expression of NCAPG significantly correlated with the phosphorylation of SRC (Tyr416) and elevated nuclear localization of STAT3 in BC samples (Fig. 7a, b). Moreover, western blotting and EMSA assay proved that NCAPG expression promoted the phosphorylation of SRC (Tyr416) (*r* = 0.831, *P* = 0.002) and STAT3 transcriptional activity (*r* = 0.804, *P* = 0.003) (Fig. 7c, d). Overall, these results suggest that NCAPG induces trastuzumab resistance in HER2+ BC by activation of SRC/STAT3 signaling.

## Discussion

Trastuzumab was the first anti-HER2 agent to be approved by the US Food and Drug Administration (FDA) and remains the backbone for treatment of HER2+ BC with the high selectivity^23^. Although trastuzumab is well tolerated, there is increasing clinical data showing trastuzumab resistance in HER2+ BC therapy^8,9^. Although trastuzumab combined with chemotherapy drugs can improve the therapeutic efficacy of patients, the mild and moderate adverse reactions after combined chemotherapy significantly increased^24,25^. Moreover, with the occurrence of trastuzumab resistance, the response duration of combined treatment is only 5–9 months^26^. New drugs are developed to overcome trastuzumab resistance in HER2+ BC, such as lapatinib and pertuzumab, etc. However, due to the single target, limited benefit or the dependency of trastuzumab, most of the alternative drugs cannot completely replace trastuzumab in efficacy and safety^27,28^. Trastuzumab is still the best first-line drug for HER2+ BC patients.

Trastuzumab blocks dimerization and activation of the HER2 receptor by binding the extracellular domain of HER2, inhibits shedding of the HER2 extracellular region to inactivate multiple intracellular signaling pathways, and mediates antibody-induced cytotoxicity to inhibit the proliferation of tumor cells^29,30^. Previous research found that effective interruption of the binding between trastuzumab and HER2 is one of the most common mechanisms of trastuzumab resistance^31^. The constitutive activation of p95HER2, the truncated form of the HER2 receptor, and high expression of membrane-related glycoprotein mucin 4 (MUC4) were both found to inhibit the binding of trastuzumab to HER2^32,33^. However, even if trastuzumab binds effectively to the HER2 extracellular segment, drug resistance can still occur through intracellular activation of signal pathways, resulting in tumor progression^34^. In line with this, our study found that the intracellular oncogene NCAPG is significantly overexpressed in HER2+ BC, particularly in patients’ samples and BC cell lines which are trastuzumab-resistant. Upregulation of NCAPG indicates poor survival and relapse in HER2+ trastuzumab-resistant BC, while knockdown of NCAPG enables re-sensitization of resistant HER2+ BC cells to trastuzumab.

NCAPG has been previously reported as a tumor-promoting gene and facilitates tumor progression. It was previously found that NCAPG promotes proliferation, metastasis, and recurrence in HCC^12^. RNA sequencing revealed that overexpression of NCAPG can induce cell proliferation and inhibit apoptosis via activation of the PI3K-AKT signaling pathway in HCC^17^. Upregulated NCAPG also correlated positively with the cell proliferation and poor survival in BC as well as the poor disease-free survival and advanced clinical stage in castration-resistant prostate cancer^14,18,19^. Silencing NCAPG was shown to enhance the effect of temozolomide on inhibition of cell proliferation in pediatric high grade gliomas^13^. However, to our knowledge, no in-depth study investigating NCAPG in BC drug resistance has been carried out. Strikingly, NCAPH, another subunit of condensin 1, which has also been shown to be a tumor-promoting factor, is expressed in hormone-sensitive prostate cancer and involved in resistance to castration^35^. Besides, overexpression of NCAPH has been found to be associated with carboplatin resistance in ovarian cancer^36^. Recently, Shimomura et al. revealed that NCAPH confers platinum resistance to oral squamous cell carcinoma cells, and NCAPG was found to interact with NCAPH^37^. These studies indicated a potential role of NCAPG in conferring drug resistance. Consistently, our study demonstrated that expression of NCAPG is increased in trastuzumab-resistant BC, and NCAPG overexpression promotes the proliferative and anti-apoptotic cell features, while silencing NCAPG re-sensitized the response to trastuzumab in trastuzumab-resistant HER2+ BC.

Previous studies demonstrated that SRC is elevated and constitutively activated in multiple cancers containing breast, colon, lung, and pancreas^38^. Extracellular signals such as PDGFR, HER family members (HER1; HER2 and HER3), and IGF1R can phosphorylate SRC on Tyr416, which is followed by activation of MAPK, PI3K, and STAT3-signaling pathways and results in cell differentiation and proliferation^39,40^. The activation of the SRC, PI3K/AKT, and STAT3 pathway can facilitate the proliferation and growth of tumor cells by upregulating the expression of Myc, CyclinD1, and CDC2; inhibit apoptosis by upregulating BCL-xL, Survivin, and Mcl; promote metastasis by upregulating FAK, p130Cs, MMP2, and MMP9; and accelerate tumor angiogenesis by upregulating VEGF and HIF1α^41–43^. These biological processes are all important mechanisms of trastuzumab resistance in BC^44^. It has been shown that SRC activation confers resistance to the HER2/EGFR tyrosine kinase inhibitor lapatinib^45^, while knockdown of SRC was found to sensitize response to PI3Kα inhibitor alpelisib in ER+ BC cells^46^. Additionally, the constitutive activation of STAT3 was reported to be accompanied by overexpression and activation of SRC in BC, resulting in chemotherapeutic resistance^47^. SRC inhibitors were shown to reduce STAT3 activity and sensitize the response to Taxol in HER2-overexpressing BC^48^. All these studies suggest SRC/STAT3 signaling could be a potential target of trastuzumab-resistant BC. Herein, we found that NCAPG confers trastuzumab resistance by activating the SRC/STAT3-signaling pathway, which demonstrates a novel mechanism of trastuzumab resistance and provide the potential target for therapy. The further mechanism of NCAPG activating the SRC/STAT3 is necessary and has been under our investigation. It has been reported that HER2-induced secretion of IL-6 can act in an autocrine fashion in human tumor cells causes STAT3-mediated gene expression and signaling activation and facilitates oncogenic growth^49^. Lu et al. found a binding site of STAT3 to HER2 promoter and observed a stimulatory effect both on HER2 mRNA and protein expressions in MCF-7 cells stably expressing STAT3, suggesting that STAT3 upregulated HER2 expression^50^. Collectively, HER2 might take part in the regulation of NCAPG to SRC/STAT3 and a potential positive feedback loop regulation mechanism may exist in this regulatory network.

In conclusion, our study revealed that NCAPG promotes trastuzumab resistance, at least in part via SRC/STAT3 pathway activation. Our results demonstrate a critical role for NCAPG in conferring trastuzumab resistance and suggest that NCAPG may be a potential therapeutic target against trastuzumab resistance in HER2+ BC.

## Materials and methods

### Cell lines and cell culture

The human HER2+ BC cell lines, SKBR3 and BT474, were purchased from ATCC (Manassas, VA, USA). SKBR3 cells were cultured in McCoy’5A (GIBCO, Grand Island, NY) containing 10% fetal bovine serum (FBS) (HyClone, Logan, UT), and BT474 cell lines were cultured in DMEM (GIBCO) medium supplemented with 10% FBS in a humidified environment with 5% CO_2_ at 37 °C. All cell passaging or performing experiments were performed while cells reached 50–80% density. 1 × 10^6^ cells were passed into 10 cm culture dishes and incubated for 24 h ahead of trastuzumab exposure. The cells were continuously treated with trastuzumab in concentration of 10, 50, 100 μg/mL. The process was conducted for ~6 months to induce trastuzumab-resistant SKBR3/TR and BT474/TR cells. Trastuzumab is a gift from the Sun Yat-sen University Cancer Center. The SRC inhibitor, PP2 (10 μM), was purchased from Selleck Chemicals (Houston, TX).

### BC biopsies

This study collected 12 BC biopsies from patients who had been treated with trastuzumab at the Sun Yat-sen University Cancer Center from 2002 to 2007. The biopsies were acquired prior to the initiation of trastuzumab. Tumor response to therapy defined as either complete response (CR), partial response (PR), stable disease (SD), or progressive disease (PD), which was notarized by computed tomography and evaluated in line with the Response Evaluation Criteria in Solid tumors (RECIST) guidelines. The six cases with CR or PR were defined as trastuzumab sensitivity, whereas the other six cases with SD or PD were considered trastuzumab resistance. This study was licensed by the Institutional Research Ethics Committee, and the informed consents were obtained before using the specimens.

### IHC assay

IHC staining was performed on the 103 paraffin-embedded BC tissue sections with trastuzumab treatment, using anti-NCAPG, anti-p-SRC (Tyr416), and anti-p-STAT3 (Tyr705) antibodies. Two independent pathologists, blinded to the histopathological features and patient data, separately reviewed and scored the degree of immunostaining of the sections. IHC-staining intensity was scored: strong (brown): +3; moderate (yellow brown): +2; weak (light yellow): +1; and negative (no staining): 0. The proportion of tumor cells was evaluated as follows: 0 (no staining tumor cells), 1 (<10% staining tumor cells), 2 (10–50% staining tumor cells), and 3 (>50% staining tumor cells). The staining index was scored as staining intensity by the proportion of tumor cells. The cut off value was the median value 3. Staining index score 0, 1, 2, 3 were defined as low expression, whereas those that scored 4, 6, 9 were considered as high expression. Specimens appearing >10% nuclear expression were considered nuclear positive.

### Constructs, transfection, and retroviral infection

The plasmids pLV-EF1A-hNCAPG and pLV[shRNA]-U6-hNCAPG (shRNA#1 and shRNA#2) were purchased from VectorBuilder (VectorBuilder Inc., Guangdong, China). Lipofectamine 3000 reagent (Invitrogen, Carlsbad, CA) was used for transfection according to the manufacturer’s instructions. Stable cell lines expressing NCAPG or NCAPG-shRNAs were selected over 10 days using 0.5 μg/mL puromycin. Cell lysates were prepared to RNA/protein extraction for further experiments.

### RNA extraction, reverse transcription, and qPCR

Total RNA was isolated using Trizol reagent (Invitrogen). RNA was quantified using a NanoDrop ND-1000. Reverse transcription-PCR was implemented using a SuperScript One-Step RT-PCR with Platinum Taq (Invitrogen) and 1 μg RNA in a Bio-Rad MyCyler (Bio-Rad, Hercules, CA). Quantitative real-time PCR (qPCR) was conducted using the FastStart Universal SYBR Green Master (Roche, Toronto, ON, Canada) with a Bio-Rad CFX96 Real Time System C1000 Cycler (Bio-Rad). The levels of gene expression were normalized to the housekeeping gene, GAPDH, using CFX Manager V2 software.

### Western blotting analysis

RIPA lysis buffer supplemented with a Protease/Phosphatase Inhibitor Cocktail (Cat# 5872, Cell Signaling Technology, Danvers, MA) was used to extract from both the tissue and cell samples. The protein was quantified using a bicinchoninic acid (BCA) assay (Pierce, Rockford, IL) according to the manufacturer’s instructions. The same amount (30 μg) protein of each sample was separated using SDS–PAGE and transferred to polyvinylidene difluoride membranes (Roche). The membranes were blocked with 5% nonfat milk in TBST (0.1% Tween-20) for 1 h at room temperature, and subsequently incubated with the appropriate primary antibodies overnight at 4 °C. After washing three times in TBST, the membranes were incubated with horseradish peroxidase (HRP)-conjugated secondary antibodies (1:5000, Cat# ab7090, Cat# ab97040, Abcam, Cambridge, MA) for 1 h at room temperature. The following antibodies were used: anti-NCAPG rabbit polyclonal antibody (1:1000, Cat# 24563-1-AP, PROTEINTECH, Hubei, China), and anti-p84 (Cat# ab102684, Abcam), anti-STAT3 (Cat# ab119352, Abcam), anti-pSTAT3 (Tyr705, Cat# ab76315, Abcam), and anti-SRC (Cat# 2109), anti-p-SRC (Tyr416, Cat#2101), anti-Cyclin D1 (Cat# 55506), anti-BCL2 (Cat# 15071), anti-GAPDH (Cat# 5174), and anti-α-Tubulin (Cat# 2125) (1:1000, Cell Signaling Technology).

### Colony formation assay

Cells were seeded into a six-well culture plates at a density of 1.0 × 10^3^ cells and grown in adaptive medium in a humidified environment with 5% CO_2_ at 37 °C for 18 h, then replaced the medium by a fresh medium containing 10 μg/mL trastuzumab for incubating for 14 days. After the culture period, discarded the culture and fixed the cells by 4% polyoxymethylene for 30 min then stained cells with crystal violet, and counted under an inverted microscope.

### Annexin V-FITC/propidium iodide (PI)-stained assay

Cells were seeded into a six-well plate at a density of 60% for analyzing the cellular apoptotic rate using flow cytometry according to the standard manufacturer protocol (MACS Miltenyi Biotec, Germany). The cells were trypsinized, washed with ice-cold PBS, and centrifuged at 1000×*g* for 5 min, followed by re-suspension in binding buffer at a density of 1.0 × l0^6^ cells/mL. Subsequently, the cells were incubated with Annexin V-isothiocyanate fluorescein and PI (BD, CA) for 25 min at 4 °C in dark. After that, the stained cells were analyzed using Cytomics FC500 (Beckman Coulter, Miami, FL) at an excitation wavelength of 488 nm. Apoptotic cells were the Annexin V-positive cells.

### Terminal transferase dUTP nick end labeling (TUNEL) assays

The indicated cells and tissues were fixed with paraformaldehyde. The TUNEL Assay Kit was used to assess the cell apoptosis according to the manufacturer’s instruction (KeyGEN, Guangdong, China). In brief, cells or tissues were fixed in 4% paraformaldehyde for 30 min at room temperature, washed three times with PBS and permeabilized with 0.1% Triton-X 100 for 5 min at room temperature. Then the samples were stained with Streptavidin-TRITC under the action of TdT enzyme for 30 min at 37 °C, washed three times with PBS, and counterstained cell nuclei with DAPI. The images were obtained with fluorescence microscope (Leica, Buffalo Grove, IL).

### Tumor xenografts

All animal experiments were approved by the Institutional Animal Care and Use Committee of Sun Yat-sen University. Animals were randomly divided into groups and the experiments were performed independently and blindly. Briefly, 2 × 10^6^ cells (SKBR3/TR-Scramble, SKBR3/TR-shRNA#1, SKBR3/TR-shRNA#2) were subcutaneously injected into the mammary fat pad of 4-week-old female BALB/c nude mice (19–22 g). When the average size of tumor reached 100 mm^3^, the mice were injected with trastuzumab (10 mg/kg, once a week) intraperitoneally for 4 weeks. The mice weight was measured every week. The tumor volume was calculated using the equation: (*L* × *W*^2^)/2. The mice were sacrificed after 4-week treatment, and the tumors were harvested, weighed, and photographed. Serial 6.0 μm sections were sliced and underwent TUNEL (KeyGEN) staining to analyze the apoptosis rate.

### Luciferase reporter assays

Cells were seeded in triplicate into 24-well plates at a density of 5 × 10^4^. 500 ng STAT3 luciferase reporters plus 5 ng pRL-TK Renilla plasmid (Promega, Madison, WI) were transfected into indicated cells using Lipofectamine 3000 reagent (Invitrogen) according to the manufacturer’s instruction. The luciferase and Renilla signals were measured at 36 h after transfection using a Dual Luciferase Reporter Assay Kit (Promega). The relative luciferase activity was calculated by normalizing to the Renilla signal.

### Immunofluorescence (IF) staining

Cells (5 × 10^4^) were cultured on slides for 24 h, washed with PBS for three times and treated with 1% Triton X-100. The cells were stained with anti-STAT3 (Cell Signaling Technology), for 2 h at 4 °C, washed with PBS, and incubated with a TRITC-conjugated secondary antibody (1:100, Cell Signaling Technology) at 37 °C for 1 h. The cell nuclei were visualized by counterstaining with DAPI (25 ng/mL, Cat#, D9542, Sigma, St. Louis, MO). The images were obtained with a confocal laser-scanning microscope (Carl Zeiss, Oberkochen, Germany).

### Electrophoretic mobility shift assay (EMSA)

EMSA was performed according to the manufacturer’s protocol using a LightShift Chemiluminescent EMSA kit (Pierce Biotechnology, Rockford, IL). DNA probes containing specific binding sites were used: STAT3: sense, 5′-GATCCTTCTGGGAATTCCTAGATC-3′, antisense, 5′-GATCTAGGAATTCCCAGAAGGATC-3′; OCT-1 (used as the negative control): sense, 5′-TGTCGAATGCAAATCACTAGAA-3′, antisense, 5′-TTCTAGTGATTTG CATTCGACA-3′.

### Statistical analysis

Data were analyzed using the SPSS version 19.0 statistical software package. The statistical tests used to analyze the data included a log-rank test, *χ*^2^ test, Spearman-rank correlation test, and Student’s *t* test (two-sided). The data are presented as the mean ± SD. A threshold of *P* < 0.05 indicated statistical significance.

## Supplementary information

Supplementary Information

Supplementary Information 2

Supplementary Information 3

Supplementary Information 4

Supplementary Information 5

Supplementary Information 6

Supplementary Information 7

Supplementary Information 8
