# Supplementary material for: NCAPG confers trastuzumab resistance via activating SRC/STAT3 signaling pathway in HER2-positive breast cancer
Source: Cell Death Dis. 2020 Jul 18;11(7):547. doi: 10.1038/s41419-020-02753-x (PMC7368860; doi:10.1038/s41419-020-02753-x)
Supplement: Supplementary file 1 — Supplementary Information [file 41419_2020_2753_MOESM1_ESM.doc]

**Supplementary figure legend**

**Fig. S1** The expression of NCAPG in TCGA. (a) Quantification of NCAPG mRNA expression in 1095 breast cancer specimens vs. 113 normal breast tissues from TCGA (*P* < 0.001). (b) Quantification of NCAPG mRNA expression in 558 HER2- *vs.* 162 HER2+ breast cancer specimens from TCGA (*P* < 0.001).

**Fig. S2** qPCR and western blotting results of NCAPG expression in trastuzumab-resistant SKBR3 and BT474 BC cell lines compared with their parental cells.

**Fig. S3** Kaplan–Meier analysis of overall survival, distance metastasis-free survival, relapse-free survival for patients with breast cancer in the data of Kaplan Meier-plotter ([*http://kmplot.com*](http://kmplot.com/)).

**Fig. S4** The expression of NCAPG in indicated SKBR3/TR and BT474/TR cells (**a**) and xenograft tumor tissues (**b**), measured by western blotting.

**Fig. S5** The expression of NCAPG in indicated SKBR3 and BT474 cells, measured by western blotting.

**Fig. S6** The nuclear expression of STAT3 in indicated breast cancer cell lines, measured by western blotting.
