# Supplementary material for: NCAPG confers trastuzumab resistance via activating SRC/STAT3 signaling pathway in HER2-positive breast cancer
Source: Cell Death Dis. 2020 Jul 18;11(7):547. doi: 10.1038/s41419-020-02753-x (PMC7368860; doi:10.1038/s41419-020-02753-x)
Supplement: Supplementary file 2 — Supplementary Information 2 [file 41419_2020_2753_MOESM2_ESM.doc]

**Supplementary Tables**

**Supplementary Table S1**. Clinicopathological characteristics of studied patients and expression of NCAPG in breast cancer

| **Factor** | **No.** | **(%)** |
| --- | --- | --- |
| **Age (years)** |  |  |
| ≤ 48 | 53 | 51.5 |
| >48 | 50 | 48.5 |
| **Clinical stage** |  |  |
| I | 14 | 13.6 |
| II | 44 | 42.7 |
| III | 42 | 40.8 |
| IV | 3 | 2.9 |
| **T classification** |  |  |
| T_1_ | 24 | 23.3 |
| T_2_ | 57 | 55.3 |
| T_3_ | 16 | 15.5 |
| T_4_ | 6 | 5.8 |
| **N classification** |  |  |
| N_0_ | 39 | 37.9 |
| N_1_ | 29 | 28.2 |
| N_2_ | 20 | 19.4 |
| N3 | 15 | 14.6 |
| **M classification** |  |  |
| No | 100 | 97.1 |
| Yes | 3 | 2.9 |
| **Relapse status**  No | 68 | 66.0 |
| Yes | 35 | 34.0 |
| **Vital status** |  |  |
| Alive | 61 | 59.2 |
| Dead | 42 | 40.8 |
| **Expression of NCAPG** |  |  |
| Low expression | 68 | 66.0 |
| High expression | 35 | 34.0 |

**Supplementary Table S2**. Correlation between the clinicopathological features and expression of NCAPG

| **Patient characteristics** | | **NCAPG expression** | | ***P*-value** |
| --- | --- | --- | --- | --- |
|  |  | **Low** | **High** |  |
| **Age (years)** | ≤ 48 | 43 | 10 | 0.009 |
|  | >48 | 25 | 25 |  |
| **Clinical stage** | I | 14 | 0 | 0.003 |
|  | II | 28 | 16 |  |
|  | III | 26 | 16 |  |
|  | IV | 0 | 3 |  |
| **T classification** | T_1_ | 21 | 3 | 0.022 |
|  | T_2_ | 34 | 23 |  |
|  | T_3_ | 11 | 5 |  |
|  | T_4_ | 2 | 4 |  |
| **N classification** | N_0_ | 33 | 6 | 0.160 |
|  | N_1_ | 12 | 17 |  |
|  | N_2_ | 13 | 7 |  |
|  | N3 | 10 | 5 |  |
| **M classification** | No | 68 | 32 | 0.014 |
|  | Yes | 0 | 3 |  |
| **Relapse status** | No | 53 | 15 | <0.001 |
|  | Yes | 15 | 20 |  |
| **Vital status** | Alive | 50 | 11 | <0.001 |
|  | Dead | 18 | 24 |  |

**Supplementary Table S3**. Univariate and multivariate analysis of different prognostic parameters in patients with breast cancer by Cox-regression analysis

|  | Univariate analysis | | |  | Multivariate analysis | | |
| --- | --- | --- | --- | --- | --- | --- | --- |
|  | *P* | Relative risk  [Exp(B)] | 95% confidence interval |  | *P* | Relative risk  [Exp(B)] | 95% confidence interval |
| **Clinical stage** | <0.001 | 3.821 | 2.122-6.881 |  | 0.027 | 3.606 | 1.154-11.270 |
| **T classification** | 0.004 | 1.746 | 1.192-2.557 |  | 0.944 | 1.019 | 0.609-1.704 |
| **N classification** | 0.001 | 1.609 | 1.208-2.145 |  | 0.800 | 0.938 | 0.574-1.536 |
| **M classification** | <0.001 | 13.233 | 3.573-49.014 |  | 0.818 | 1.311 | 0.131-13.116 |
| **Expression of NCAPG** | <0.001 | 3.823 | 1.946-7.512 |  | 0.013 | 2.516 | 1.217-5.200 |
