# Supplementary figures and images for: NCAPG confers trastuzumab resistance via activating SRC/STAT3 signaling pathway in HER2-positive breast cancer
Source: Cell Death Dis. 2020 Jul 18;11(7):547. doi: 10.1038/s41419-020-02753-x (PMC7368860; doi:10.1038/s41419-020-02753-x)

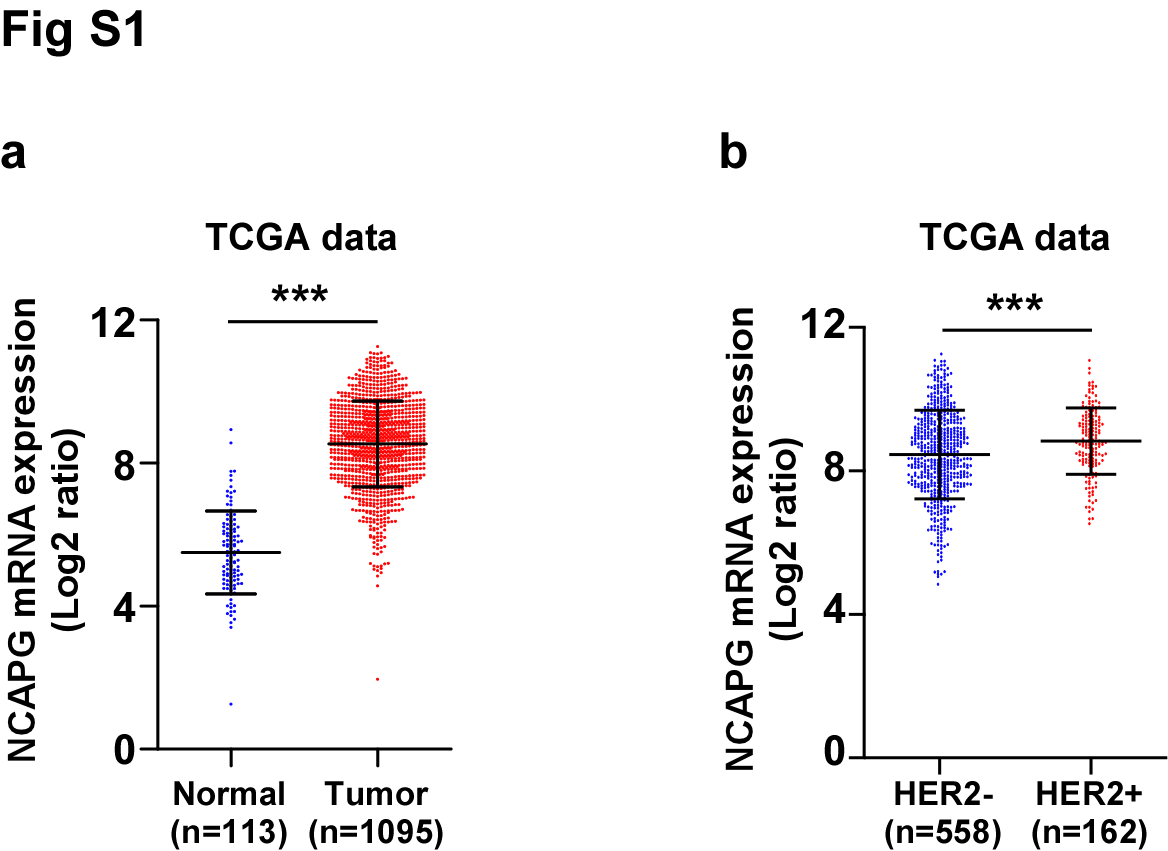

Supplement: Supplementary file 3 — Supplementary Information 3 [file 41419_2020_2753_MOESM3_ESM.tif]

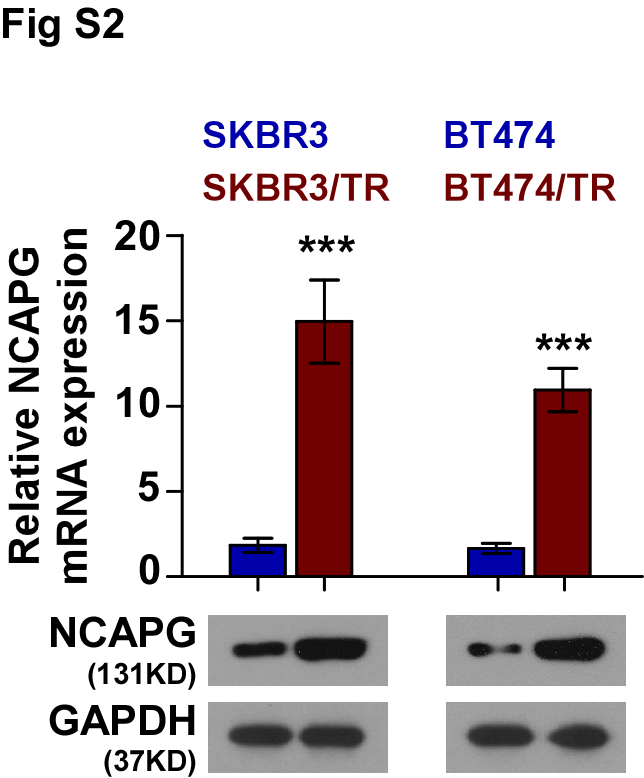

Supplement: Supplementary file 4 — Supplementary Information 4 [file 41419_2020_2753_MOESM4_ESM.tif]

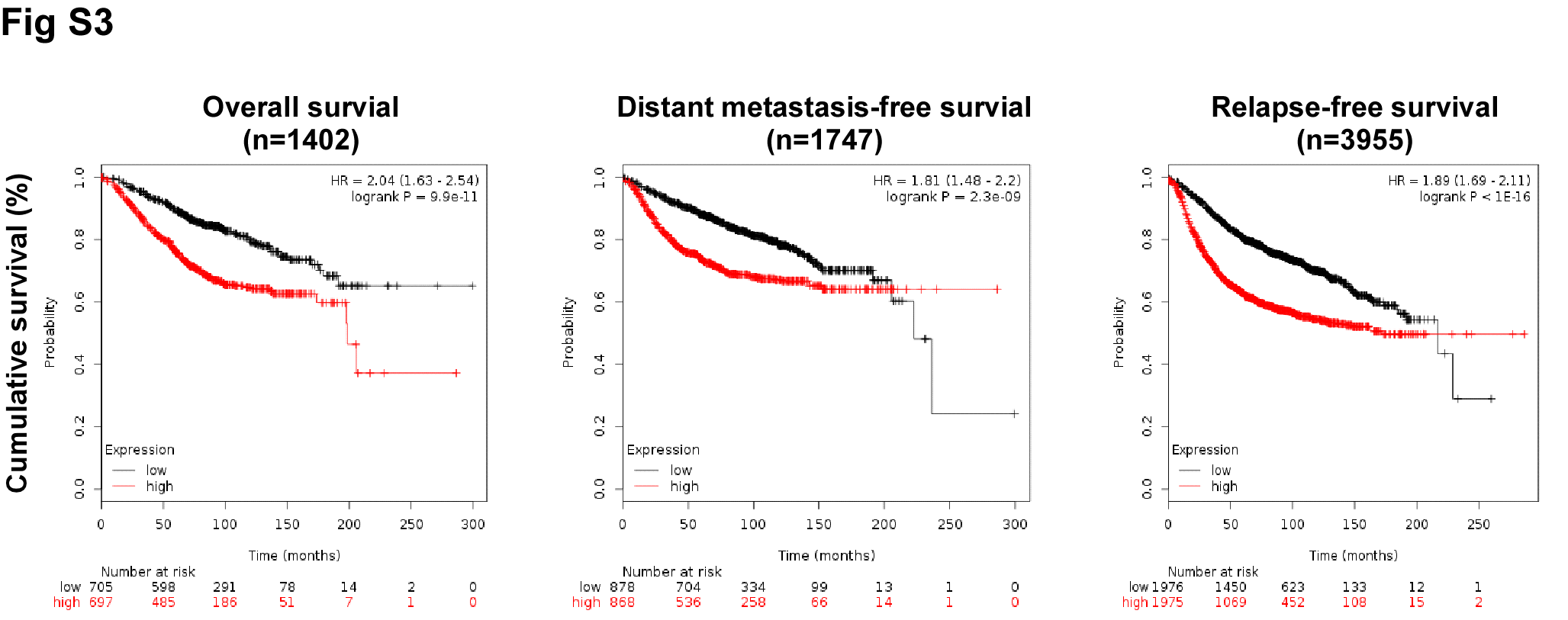

Supplement: Supplementary file 5 — Supplementary Information 5 [file 41419_2020_2753_MOESM5_ESM.tif]

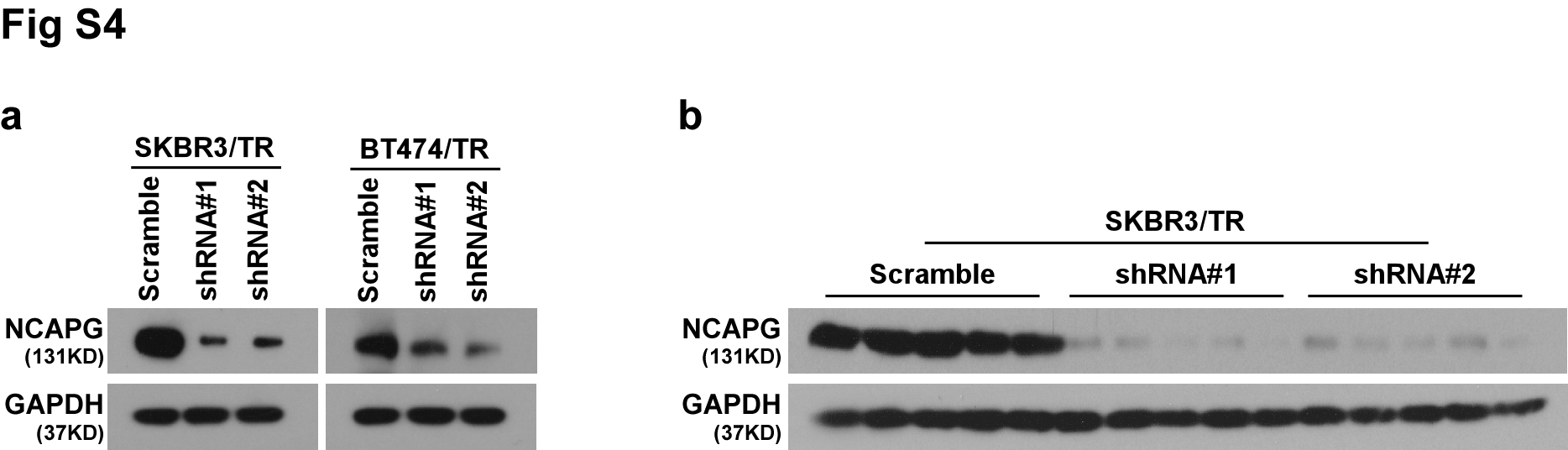

Supplement: Supplementary file 6 — Supplementary Information 6 [file 41419_2020_2753_MOESM6_ESM.tif]

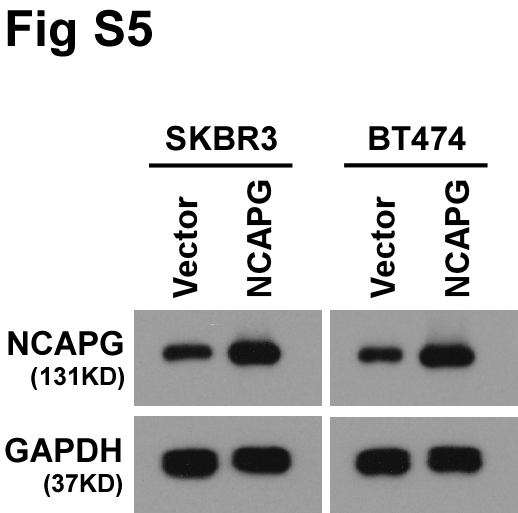

Supplement: Supplementary file 7 — Supplementary Information 7 [file 41419_2020_2753_MOESM7_ESM.tif]

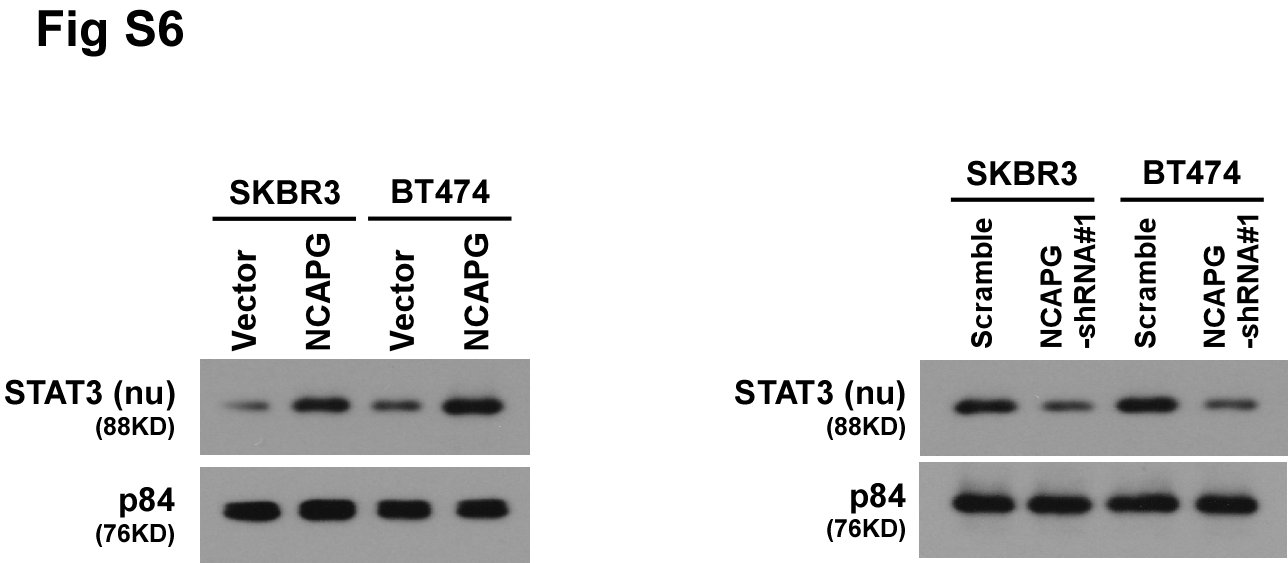

Supplement: Supplementary file 8 — Supplementary Information 8 [file 41419_2020_2753_MOESM8_ESM.tif]
